# Surmounting Cytarabine-resistance in acute myeloblastic leukemia cells and specimens with a synergistic combination of hydroxyurea and azidothymidine

**DOI:** 10.1038/s41419-019-1626-x

**Published:** 2019-05-17

**Authors:** May Levin, Michal Stark, Bluma Berman, Yehuda G. Assaraf

**Affiliations:** The Fred Wyszkowski Cancer Research Laboratory, Department of Biology, Technion-Israel Institute of Technology, Haifa, Israel

**Keywords:** Cancer therapeutic resistance, Targeted therapies, Acute myeloid leukaemia

## Abstract

Acute myeloid leukemia (AML) patients display dismal prognosis due to high prevalence of refractory and relapsed disease resulting from chemoresistance. Treatment protocols, primarily based on the anchor drug Cytarabine, remained chiefly unchanged in the past 50 years with no standardized salvage regimens. Herein we aimed at exploring potential pre-clinical treatment strategies to surmount Cytarabine resistance in human AML cells. We established Cytarabine-resistant sublines derived from human leukemia K562 and Kasumi cells, and characterized the expression of Cytarabine-related genes using real-time PCR and Western blot analyses to uncover the mechanisms underlying their Cytarabine resistance. This was followed by growth inhibition assays and isobologram analyses testing the sublines’ sensitivity to the clinically approved drugs hydroxyurea (HU) and azidothymidine (AZT), compared to their parental cells. All Cytarabine-resistant sublines lost deoxycytidine kinase (dCK) expression, rendering them refractory to Cytarabine. Loss of dCK function involved dCK gene deletions and/or a novel frameshift mutation leading to dCK transcript degradation via nonsense-mediated decay. Cytarabine-resistant sublines displayed hypersensitivity to HU and AZT compared to parental cells; HU and AZT combinations exhibited a marked synergistic growth inhibition effect on leukemic cells, which was intensified upon acquisition of Cytarabine-resistance. In contrast, HU and AZT combination showed an antagonistic effect in non-malignant cells. Finally, HU and AZT synergism was demonstrated on peripheral blood specimens from AML patients. These findings identify a promising HU and AZT combination for the possible future treatment of relapsed and refractory AML, while sparing normal tissues from untoward toxicity.

## Introduction

Acute myeloid leukemia (AML) is a hematopoietic malignancy of the myeloid lineage displaying rapid proliferation and accumulation of undifferentiated myeloid cells in the bone marrow, thereby interfering with the production and maturation of normal blood cells. Since 1973, AML treatment relies on the anchor pro-drug Cytarabine (cytosine arabinoside, Ara-C), a cytidine analog^1–3^. However, whereas 70–80% of AML patients achieve remission following induction chemotherapy, 80% of them relapse for which no salvage regimen currently exists^2–4^.

Cytarabine uptake via equilibrative nucleoside transporters (ENTs)^5^ and concentrative nucleoside transporter 3 (CNT3)^6^, is followed by three consecutive phosphorylation steps resulting in the cytotoxic metabolite Ara-CTP^1^. The rate-limiting enzyme in Cytarabine phosphorylation is deoxycytidine kinase (dCK), a central enzyme in the nucleoside salvage pathway (NSP)^7–9^, which also phosphorylates the naturally occurring deoxycytidine, deoxyadenosine and deoxyguanosine to their monophosphate form^8^. Loss of dCK activity is a *bona fide* mechanism of Cytarabine resistance in AML patients and model tumor cell lines^10–17^, leading to cross-resistance to various nucleoside analog pro-drugs^13,18–20^ requiring activation via phosphorylation. Cytarabine resistance may also include impaired activity of ENTs^13,17,21^, and upregulation of cytidine deaminase (CDA)^22^. Thus, the high relapse rate due to Cytarabine resistance calls for novel therapeutic modalities. Although, different cytotoxic agents were tested for relapsed AML, usually in combination with Cytarabine, there was no substantial improvement in success rates^2^. These include ribonucleotide diphosphate reductase (RNR) inhibitors, which increase Ara-CTP levels in AML blasts^2^; however, these nucleoside analogs also rely on phosphorylation by dCK, rendering them ineffective towards Cytarabine-resistant clones lacking dCK activity^19,23,24^. Since loss of dCK abolishes NSP, AML cells are more dependent on the de novo nucleotide synthesis pathway (DNSP). Hence, the RNR inhibitor hydroxyurea (HU), which is clinically used to manage myeloproliferative disorders, sickle cell disease, and AIDS^25–28^, was previously suggested for AML treatment. Enhancement of Cytarabine toxicity by HU was demonstrated in leukemia cell lines^29,30^.

The goal of the current study was to identify a treatment modality, which could surmount Cytarabine resistance in AML cells. We found that Cytarabine-resistant sublines displayed hypersensitivity to a combination of HU and azidothymidine (AZT), compared to parental cells; this combination exhibited a marked synergistic activity on hematopoietic cells including primary cells from AML patient specimens, which was potentiated upon acquisition of Cytarabine-resistance. In contrast, this combination showed an antagonistic effect in non-malignant cells.

## Materials and methods

### Tissue culture

Human chronic myelogenous leukemia (CML) K562 cells, cervical cancer HeLa cells, and embryonic HEK293 cells were maintained in RPMI-1640 medium (Gibco, Life Technologies, Grand Isle, NY) containing 10% fetal bovine serum, 2 mM glutamine, 100 μg/ml penicillin, and streptomycin (Biological Industries, Beit HaEmek, Israel), and kept in a humidified atmosphere of 5% CO_2_ at 37 °C. The AML cell line Kasumi-1 [genotype t(8:21) leading to AML1-ETO fusion^31^] was similarly grown in RPMI-1640 medium containing 20% fetal bovine serum.

#### Cytarabine selection

Multiple step selections with gradually increasing Cytarabine concentrations (cat. C1768, Sigma Aldrich, St. Louis, MO, USA) was performed on K562 and Kasumi cells for the establishment of drug-resistant sublines, using a starting dose of approximately twofold their original IC_50_ values (Table 1); the latter were obtained by growth inhibition assays as detailed below. K562 cells were continuously grown in 0.2 μM Cytarabine for 28 days until cells resumed their original doubling time, yielding a drug-resistant subline termed (KAR)-0.2 (K562 Ara-C resistant); at this passage (day 28 from initiation of drug selection), KAR-0.2 cells were frozen down in aliquots and thawed for any experiment that required the original cells. KAR-0.2 cells were also transferred to grow in either 0.4 or 1 μM Cytarabine as described in the supplemental scheme (Supplementary Fig. S1), resulting in the sunlines KAR-0.4 and KAR-1, respectively. Following their establishment, KAR-0.2 and KAR-1 cells were also grown in drug-free medium to evaluate the stability of their drug resistance phenotype [the subsequent cells are termed KAR-0.2(-) and KAR-1(-), respectively].

**Table 1:** Characteristics of cytarabine-resistant sublines and patient specimens

Cell line	IC_50_ values			dCK status (%)	
	Ara-C (nM)	HU (μM)	AZT (μM)	gDNA	mRNA
K562	106 ± 16 (1)	1591 ± 160 (1)	4521 ± 580 (1)	100 ± 9	100 ± 8
KAR-0.2 < 38d	2769 to 82,684 (26–780)	251 ± 23 (0.16)	1659 ± 160 (0.4)	74 ± 5	143 ± 5
KAR-0.2 (-)	918 ± 139 (8.7)	249 ± 27 (0.16)	1750 ± 96 (0.4)	N.D	97 ± 21
KAR-0.2 ≥ 38d	>100,000 (>1000)	N.D	N.D	N.D	20 ± 9
KAR-0.4	>100,000 (>1000)	122 ± 14 (0.08)	820 ± 125 (0.2)	70 ± 4	19 ± 3
KAR-1	>100,000 (>1000)	258 ± 33 (0.16)	413 ± 17 (0.1)	57 ± 7	16 ± 5
Kasumi	38 ± 12 (1)	158 ± 12 (1)	162 ± 32 (1)	100 ± 10	100 ± 8
Kas-80	>100,000 (>2632)	84 ± 3 (0.5)	104 ± 21 (0.6)	0.7 ± 0.6	0.06 ± 0.07

AML specimen	IC_50_ values			
	Ara-C (nM)	HU (μM)	AZT (μM)	Doxorubicin (nM)
Patient 1	332	>1000	2098	940
Patient 2	83	331	249	188

The values in parentheses are the ratio over parental cells. *ND* Not determined

Kasumi cells were continuously grown in 80 nM Cytarabine for 21 days until they regained their original doubling time, resulting in a drug-resistant subline stably growing in 80 nM Cytarabine termed Kas-80.

### Patients’ specimens

Adult AML patients’ specimens studied in the current paper were previously derived as part of the routine clinical management at the Rambam Health Care Campus (Haifa, Israel). The use of the samples was approved by the IRB committee (study number 2902) following informed consent by the patients in accordance with the Declaration of Helsinki. White blood cells were isolated from peripheral blood by standard Ficoll-Hypaque (Sigma Aldrich) gradient density centrifugation. The resultant cells were cryopreserved in aliquots in fetal calf serum supplemented with 10% DMSO until analysis. Two samples were chosen according to the following criteria: high white blood cell (WBC) count ≥15,000 per µicroliter and high blast percentage ≥80%, in order to allow for sufficient cells for the isobologram study and additional analyses. Patients 1 and 2 were, respectively, a 58-years-old male with a refractory disease (recurrence during the first month of induction therapy) and a 45-years-old female at diagnosis.

### Growth inhibition assays and isobologram analysis

Hydroxyurea (HU, cat. H8627), azidothymidine (AZT, cat. A2169), and doxorubicin (DOX, cat. D1515) were purchased from Sigma Aldrich. For the analysis of cell lines, cells continuously growing in Cytarabine-containing medium were grown in drug-free medium for 3 days prior to experiments. 2 × 10^4^ cells/well were seeded in 96-well plates, and supplemented with increasing drug concentrations for 72 h. For primary AML specimens, ~40 × 10^6^ cells were thawed and washed twice with RPMI-1640 medium containing 20% FBS. Cells were then resuspended in *Hematopoietic Cell Karyotyping Medium* (Biological Industries, cat. 01-200-1) at a density of 1.4 × 10^6^ cells/ml and left to equilibrate for two hours in a humidified atmosphere of 5% CO_2_ at 37 °C. Cells (1.5 × 10^5^ cells/well) were then seeded in the same medium in 96-well plates and supplemented with the appropriate drug concentrations. For single-drug assays, drug concentrations were increased by a third of a log (0–100 μM Cytarabine, 0–30 mM HU, 0–10 mM AZT, and 0–30 µM DOX). For the drug combination treatments, cells were co-incubated with linearly increasing HU and AZT concentrations (0–3 and 0–5 mM, respectively) creating a matrix of drug combinations (AZT concentration increased throughout the lines while HU concentration increased throughout the columns). After 72 h of drug incubation, cell viability was evaluated using an XTT cell proliferation kit according to the instructions of the manufacturer (Biological Industries). The IC_50_ values determined for each cell line represent the drug concentration exerting 50% growth inhibition compared to drug-free control.

Isobologram analysis was performed to evaluate HU and AZT drug–drug interactions. This method was implemented by using experimental dose matrix data to draw a contour, as a function of the drug concentrations at which their combination achieved 50% growth inhibition (i.e., IC_50_ pairs). This contour was evaluated against the *Loewe Additivity line*^32^ connecting the two axis intersection points (i.e., the IC_50_ values of each drug alone), where a concave graph indicates synergism of the drug combination, and a convex graph demonstrates antagonism. The *Combination Index (CI)*^32^ was calculated for each IC_50_ pair and used for comparison between cell lines, where a CI value of 1 indicates additivity. Each assay was performed with cell lines in three independent experiments in triplicates, while the assays with patients’ samples were performed once in triplicates.

### RNA and genomic DNA purification, and cDNA synthesis

Cells at the mid-log phase of growth were harvested for RNA and genomic DNA (gDNA) isolation using the TRI Reagent according to the manufacturer’s instructions (Sigma Aldrich). For cycloheximide (CHX) treatment, K562 or KAR-1 cells were seeded at a density of 2.5 × 10^5^/ml and incubated for 2 h in the presence of 100 μg/ml CHX (cat. C7698, Sigma Aldrich). Following incubation, cells were harvested for cytosolic and nuclear RNA isolation: cells were sedimented by centrifugation at 800xg for 5 min at room temperature (RT) and resuspended in ice-cold RNA-A buffer [10 mM Tris pH = 7.5, 150 mM NaCl, 1.5 mM MgCl_2_, 0.65% NP-40 in diethyl-pyrocarbonate (DEPC)-treated water] freshly supplemented with 100 U/ml RNase inhibitor (New England BioLabs, Ipswich, MA, USA). Cells were then lysed by vortex and the nuclei were sedimented by centrifugation at 800xg for 5 min at 4 °C. While nuclear RNA was isolated from the pellet using *TRI Reagent* as described above, cytosolic RNA was purified from the supernatant as follows: the supernatant was transferred to a new tube containing an equal volume of RNA-B buffer (10 mM Tris pH = 7.5, 7 M urea, 1% SDS, 350 mM NaCl, 10 mM EDTA in DEPC-treated water) and 2-volumes of phenol:chloroform:isoamylalcohol mixture (25:24:1, Sigma Aldrich), vortexed for 10 s and centrifuged at 15,000 x *g* for 1 min at room temperatue (RT). The top layer was transferred to a fresh tube, after which RNA was sedimented by incubation in 95% ethanol at −20 °C for >30 min and centrifugation at 12,000 x *g* for 10 min at 4 °C. Finally, cDNA was synthesized from purified RNA samples using the high capacity cDNA reverse transcription kit (Thermo Fisher Scientific, Waltham, MA, USA) according to the manufacturer’s instructions.

### PCR and real-time PCR (RT-PCR)

PCR was performed using red load Taq master according to the instructions of the manufacturer (Larova, Gena, Germany), with 400 nM forward and reverse oligonucleotide primers (Supplementary Table 1) and 10 ng cDNA or 20 ng gDNA template per reaction (in a total volume of 25 μl). RT-PCR was performed using the perfeCTa SYBR Green SuperMix according to the manufacturer’s instructions (Quanta bio, Beverly, MA, USA) with 150 nM forward and reverse oligonucleotide primers (Supplementary Table 1) and 5 ng cDNA or gDNA template per reaction (total volume—20 μl). All gene expression levels were normalized to glucuronidase beta *(GUSB)*, and gDNA levels were normalized to folate receptor α (gFR-α). RT-PCR reactions were conducted using the 7300 Real-Time PCR System (Applied Biosystems, CA, USA) and results were analyzed with the 7300-system sequence detection software version 1.4 (Applied Biosystems). Each experiment was performed at least three times in triplicates.

### Protein isolation and Western blot (WB) analysis

For cytosolic protein extractions, cells at the mid-log phase of growth were harvested and incubated in a hypotonic buffer (10 mM HEPES pH 8, 10 mM KCl, 0.1 M EDTA, 0.1 M EGTA and 1 mM DTT, supplemented with cOmplete mini, Roche, Basel, Switzerland) for 15 min on ice, lysed with 0.5% NP-40 and centrifuged at 1000 x *g* for 5 min at 4 °C to sediment the nuclei. The supernatant containing cytosolic proteins was transferred to a new tube, while the nuclei-containing pellet was further processed for nuclear protein extraction. Nuclei were resuspended in hypotonic buffer and sonicated (Microson, amplitude 3, Misonix, Farmingdale, NY, USA) with three 5 s pulses and 10 s intervals on ice-water. Following sonication, the extracts were centrifuged at 20,000 x g for 15 min at 4 °C to remove cell debris, and the supernatant containing nuclear proteins was collected. Patients’ cells were thawed and washed twice prior to the same isolation procedure. Protein concentrations were determined using the Bio-Rad protein Assay (Bio-Rad, Hercules, CA, USA). For histone protein analysis, cells were harvested for hot lysis extractions following 24 and 48 h incubations with 500 μM AZT: cells were incubated in a hot lysis buffer (50 mM Tris-HCl pH 8.1, 10 mM EDTA and 1% SDS) for 10 min at 100 °C and sonicated with three 15 s pulses (amplitude 4) and 15 s intervals on ice-water, followed by centrifugation to remove cell debris (20,000 x *g*, 15 min, 4 °C). Protein concentration was determined using Pierce BCA protein assay kit (Thermo Fisher Scientific). Forty microgram nuclear proteins/50 μg cytosolic proteins (for dCK, CDA, or TK1 analysis), or 30 μg histone extractions (for ɣH2AX analysis) were resolved on 12.5% polyacrylamide gels and electroblotted onto Protran BA83 nitrocellulose membranes (WhatmanTM, GE, Maidstone, UK). Membranes were blocked using TBS buffer containing 20% skim milk for 1 h at RT, reacted with a primary antibody (mouse anti-dCK: sc-393098, Santa Cruz, Dallas, TX, USA; mouse anti-CDA: sc-365292, Santa Cruz; rabbit anti-TK1: ab76495, abcam, Cambridge, UK; or mouse anti-phospho-ɣH2AX (Ser139): 05–636, Millipore, Burlington, MA, USA) for 1 h at RT and washed three time for 10 min at RT with TBS supplemented with 0.5% Tween 20 (TBST) buffer. Then, membranes were reacted with a horseradish peroxidase (HRP)-conjugated secondary antibody (goat anti-mouse: 115-035-062, or goat anti-rabbit: 111-035-045, Jackson Immunoresearch, West Grove, PA, USA) for 1 h at RT, and washed three times for 10 min at RT with TBST. Enhanced chemiluminescence (ECL) detection was performed using the EZ-ECL kit according to the manufacturer’s instructions (Biological Industries, Beth-Haemek, Israel) and the ImageQuant LAS 4000 imaging system (GE Healthcare Life Sciences, Marlborough, MA, USA). Thereafter, the membranes were stripped off with a stripping buffer (0.5 M acetic acid, 0.5 M NaCl, pH = 2.6) and reprobed with a loading control: a rabbit anti-calreticulin antibody (C4606, Sigma Aldrich) was used for dCK, CDA and TK1 analysis, and a rabbit anti-H3 antibody (ab171870, abcam) for ɣH2AX analysis; followed by the HRP-conjugated goat anti-rabbit antibody. Relative band intensities were evaluated using the ImageJ software^22^.

### DNA sequencing

The full open reading frame of dCK was PCR-amplified from cDNA in two segments (primers 1 + 4 and 3 + 8, Supplementary Table 1) as described above. PCR products were purified using the Wizard PCR and gel cleanup kit (Promega, Fitchburg, WI, USA) and sequenced at Hylabs laboratories (Rehovot, Israel) with an ABI 3730xl DNA Analyzer using BigDye Terminator 1.1 Cycle Sequencing Kit (ABI).

### Statistical analyses

Statistical significance of differences was determined using a two-tailed heteroscedastic (for comparison between cell lines) or paired (for comparison between treatment and control) Student’s *t*-test. A difference was considered statistically significant if it obtained a *P*-value of ≤0.05.

## Results

### Cytarabine-resistant myeloid leukemia sublines display markedly reduced dCK expression

**Fig. 1:**
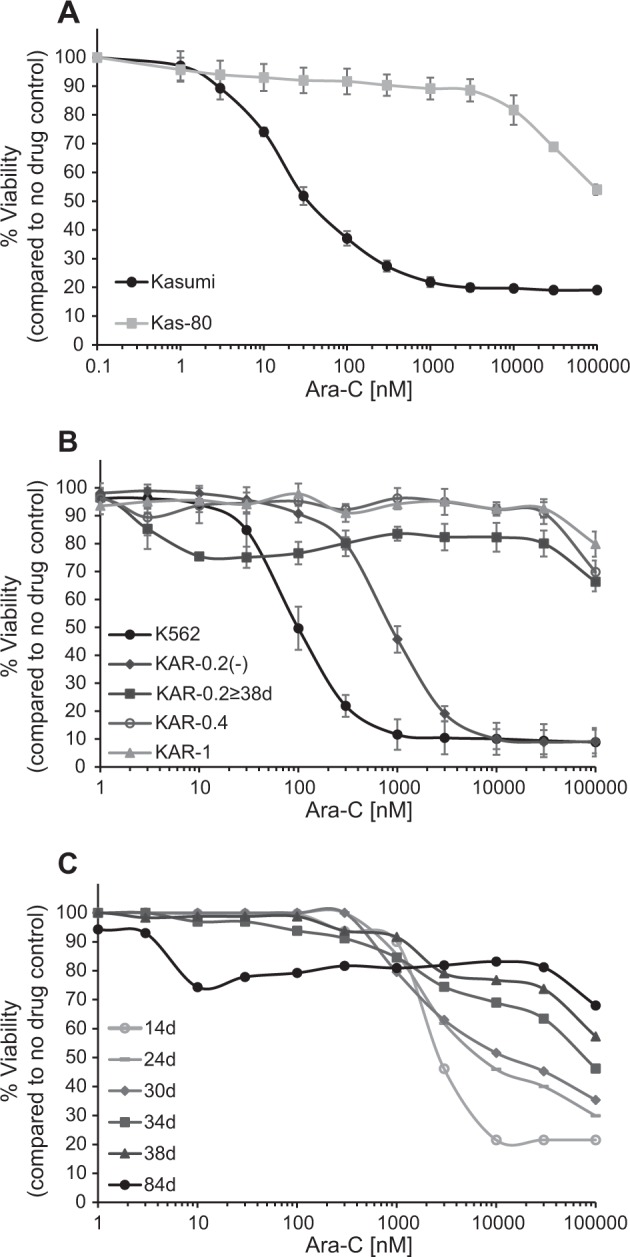
Cytarabine growth inhibition assays. Cells were incubated with increasing Cytarabine concentrations for 72 h followed by evaluation of cell viability using an XTT cell proliferation kit. **a** Cytarabine dose-dependent growth inhibition of Kasumi cells and their subline Kas-80. **b** Cytarabine dose-dependent growth inhibition of K562 cells and their sublines KAR-0.2, KAR-0.4, and KAR-1. KAR-0.2(-) represents KAR-0.2 cells continuously growing in drug-free medium since their establishment, and KAR-0.2 ≥ 38d are KAR-0.2 cells continuously growing in 200 nM Cytarabine for at least 38 days. **c** Cytarabine dose-dependent growth inhibition of KAR-0.2 cells throughout their growth period. The legend describes the number of days since the cell line was established. **a**, **b** Graphs represent the average of at least three independent experiments performed in triplicates. **c** Each graph represents a single experiment performed in triplicates

In order to explore novel treatments to overcome Cytarabine resistance in AML, we established resistant sublines by selection in increasing concentrations of Cytarabine using the two human myeloid leukemia cell lines K562 and Kasumi, as detailed in the Methods section. We then used growth inhibition assays to determine the level of Cytarabine resistance of these sublines, and found a marked increase in the IC_50_ values of all sublines (Fig. 1 and Table 1). With the exception of the K562 subline KAR-0.2, all sublines displayed a >1000-fold Cytarabine resistance, exceeding the solubility limit of the drug (i.e., 100 µM). Although stably growing in 0.2 µM Cytarabine, upon further growth under drug selective conditions, KAR-0.2 cells gradually acquired increasing levels of Cytarabine resistance (Fig. 1c and Table 1). Specifically, within 38 days of selective growth, KAR-0.2 cells, which initially displayed a 27-fold resistance, gained an additional ~37-fold increase in Cytarabine resistance exceeding 100 μM (Fig. 1c). While growth under drug selective conditions exerted a continuous acquisition of increased drug resistance levels, KAR-0.2(-) cells, which were cultured in Cytarabine-free medium since their establishment, displayed a stable, yet low level of Cytarabine resistance, throughout their drug-free growth (i.e., over 30 days, Fig. 1b and Table 1).

**Fig. 2:**
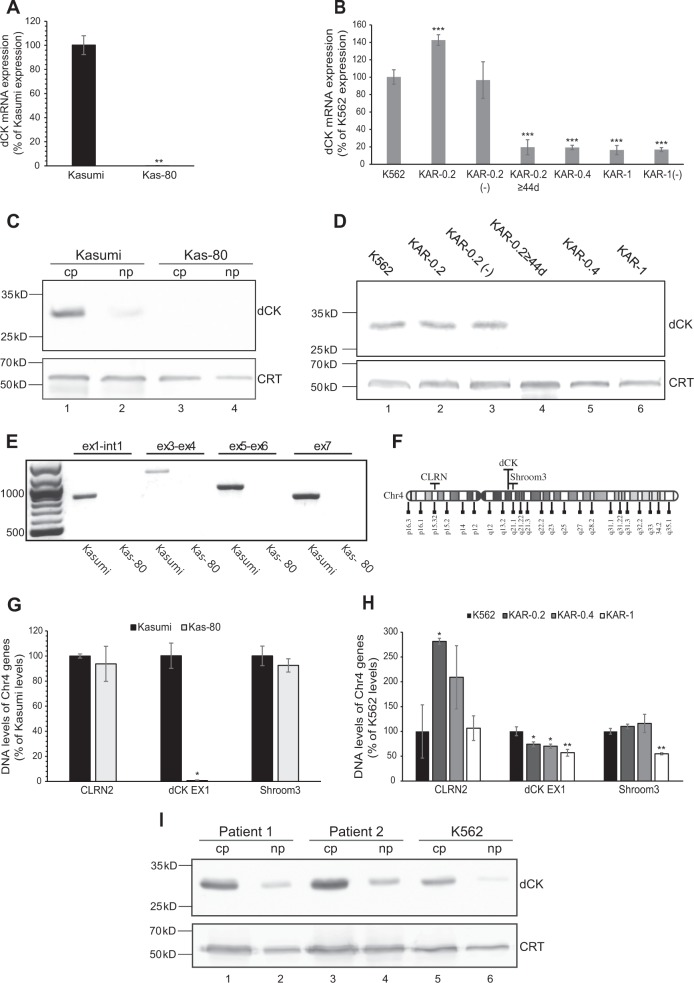
dCK expression in Kasumi, K562 and their resistant sublines, and in primary cells from AML patient specimens. Cells at mid-log phase were harvested for RNA (**a**, **b**), protein (**c**, **d**), and gDNA (**e**, **g**, and **h**) isolation. **a**, **b** RT-PCR analyses of dCK mRNA expression. **c**, **d** Representative WB analyses of dCK protein expression in cytosolic (cp) and nuclear (np) protein extracts from Kasumi and Kas-80 cells (**c**) or in cytosolic protein extractions from K562 cells and their sublines (**d**), with calreticulin (CRT) as loading control. **e** Genomic PCR analysis of dCK levels in Kasumi and Kas-80 cells throughout the dCK gene. **f** Illustration of the position of CLRN2, dCK, and Shroom3 genes on chromosome 4. **g**, **h** Genomic RT-PCR analysis of CLRN2, dCK, and Shroom3. **i** WB analyses of dCK protein levels in cp and np from primary AML patients’ white blood cells compared to K562 cells. All cell lines experiments were performed at least three times, while patients’ samples were used once due to the limited number of cells in patient specimens; RT-PCR analyses were performed in triplicates. All statistically significant changes compared to parental cells are denoted by one (*P*-value ≤ 0.01), two (*P*-value ≤ 0.001), or three (*P*-value ≤ 0.000003) asterisks

To decipher the mechanisms underlying Cytarabine resistance in the different sublines, we studied the status of proteins, which are known to contribute to Cytarabine resistance (Supplementary Fig. S2). We first verified the expression of the Cytarabine influx transporters ENT1-3 and CNT3 using RT-PCR. All sublines retained at least 74% of parental cell ENT1-3 levels and, excluding Kas-80 cells, dramatically overexpressed CNT3 (Supplementary Fig. S2A–C). Furthermore, K562 sublines exhibited a similar overexpression of an ER-resident CNT3 splicing isoform termed CNT3-Ins^33^, detected by RT-PCR using diagnostic primers (Supplementary Fig. S2C and Supplementary Table 1). We next validated dCK expression by RT-PCR and WB analysis. Kas-80 cells exhibited complete loss of dCK mRNA (Fig. 2a) and consequently undetectable dCK protein levels (Fig. 2c, lane 3). KAR-0.4 and KAR-1 cells exhibited >80% decrease in dCK transcript levels (Fig. 2b) and a complete loss of dCK protein (Fig. 2d, lanes 5–6). KAR-0.2 cells exhibited a 43% increase in dCK mRNA levels (Fig. 2b) along with the retention of parental dCK protein levels (Fig. 2d, lane 2). Remarkably, following 44 days of drug selective growth, these transcript and protein levels dropped to the poor levels observed in KAR-0.4 and KAR-1 cells. Interestingly, KAR-0.2(-) cells retained parental dCK mRNA and protein levels during the same time period (Fig. 2b, d). Recognizing that Cytarabine resistance in KAR-0.2 cells was initially dCK-independent, we also determined their CDA protein levels using WB analysis; CDA protein levels were undetectable in both parental K562 and KAR-0.2 cells (Supplementary Fig. S2E), implying that Cytarabine resistance in KAR-0.2 cells was not mediated by enhanced inactivation of the pro-drug.

### Molecular mechanisms of dCK silencing in Cytarabine-resistant sublines

To uncover the mechanisms underlying dCK silencing in our drug-resistant sublines, and considering previous reports of genomic deletions of the dCK locus in AML patients’ specimens and tumor cell lines^10,34^, we evaluated the genomic dCK levels in these sublines. PCR analysis revealed that Kas-80 cells completely lost the dCK locus as no genomic PCR product could be detected throughout the gene (Fig. 2e); this was further verified using RT-PCR (Fig. 2g). In contrast, RT-PCR analysis on gDNA from KAR-0.2, KAR-0.4, and KAR-1 cells revealed that the dCK amplicon was still retained (i.e., 74%, 70%, and 57% of parental levels, respectively, Fig. 2h). In order to evaluate whether these genomic alterations were exclusive to the dCK locus or included larger chromosomal regions, we also tested the levels of two other genes on chromosome 4: CLRN2, which is located on its short arm (Chr4p), and Shroom3, which maps to the long arm (Chr4q) downstream of the dCK gene (Fig. 2f). Kas-80 cells displayed CLRN2 and Shroom3 gDNA levels comparable to Kasumi cells (Fig. 2g); KAR-0.2 cells displayed >2-fold increase in CLRN2 genomic levels and unchanged Shroom3 levels compared to K562; KAR-0.4 cells had no significant changes in CLRN2 or Shroom3 levels, and KAR-1 cells had unchanged CLRN2 levels and a ~2-fold decrease in Shroom3 levels (Fig. 2h). These findings suggest that the genomic deletions in Kas-80, KAR-0.2 and KAR-0.4 sublines were specific to the dCK locus, while in KAR-1 cells they included a wider region of Chr4q.

### K562 sublines exhibit nonsense-mediated decay of the dCK mRNA

**Fig. 3:**
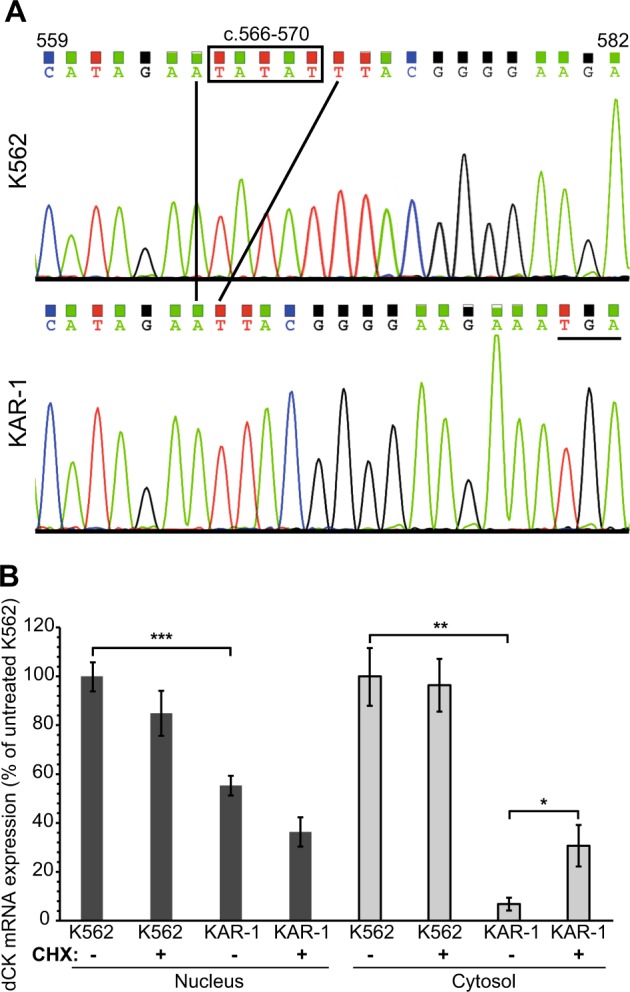
A frameshift deletion mutation in dCK leads to mRNA degradation via NMD. **a** Sequencing chromatograms of PCR-amplified dCK. Upper trace representative of K562 and KAR-0.2 cells; bottom trace representative of KAR-1, KAR-0.4, and KAR-0.2 ≥ 44d cells. The deletion is indicated by a black frame, whereas the stop codon is underlined. **b** K562 and KAR-1 cells were treated with 100 µg/ml CHX for 2 h, harvested for cytosolic and nuclear RNA purification and analyzed for dCK expression using RT-PCR. The experiment was performed four times and each RT-PCR was done in triplicates. Statistical significance is denoted by one (*P*-value = 0.02), two (*P*-value = 0.0004), or three (*P*-value = 0.0001) asterisks

While homozygous deletion can explain the complete silencing of dCK in Kas-80 cells, loss of a dCK allele in KAR-1 cells could not account for the 80% fall in mRNA levels. To further investigate dCK downregulation in Cytarabine-resistant K562 sublines, we sequenced their dCK transcript following PCR-amplification from total cell cDNA. Sequence analysis revealed a 5 bp deletion in all dCK transcripts (Fig. 3a, Δ566-570 numbered from the first ATG) from KAR-0.4, KAR-1, and KAR-0.2 cells growing more than 44 days in Cytarabine-containing medium, but not from parental K562 cells nor in the early KAR-0.2 cells displaying high dCK mRNA levels. This 5 bp deletion generated a frameshift, leading to substitution of p.YLRG190-193TGKK followed by a premature translation termination codon at position 194 (out of a total of 260 aa). The premature stop codon is located in exon 5 out of the 7 exons of dCK, over 55 nucleotides upstream of the last exon-exon junction, and therefore it should lead to degradation of the dCK transcript via nonsense-mediated mRNA decay (NMD)^35^. To validate that NMD occurs, we used cycloheximide (CHX), an eukaryotic translation inhibitor^36,37^, which indirectly attenuates the translation-dependent NMD machinery^38^. We treated KAR-1 and their parental K562 cells with 100 μg/ml CHX for 2 h, and isolated their cytosolic and nuclear RNA. First, we found that nuclear dCK RNA levels in KAR-1 cells were 55 ± 4% of parental K562 levels (Fig. 3b) in agreement with KAR-1’s genomic dCK levels, thus implying normal transcription rates of the remaining allele. Second, cytosolic dCK RNA levels in KAR-1 cells were only 7 ± 3% of parental cells (Fig. 3b). Following CHX treatment, KAR-1 cells exhibited a statistically significant 4.4-fold increase in cytosolic dCK mRNA levels, while no change was observed in K562 cells (Fig. 3b), confirming that the dCK transcript in KAR-1 cells underwent NMD. Consistently, nuclear dCK mRNA levels did not increase following CHX treatment, since the NMD pathway occurs in the cytoplasm^38^ (Fig. 3b). In agreement with the finding that dCK silencing in KAR-1 cells is a result of a genomic mutation, their dCK mRNA levels remained completely stable over 3 months of growth in drug-free medium (Fig. 2b).

### Cytarabine-resistant sublines display increased sensitivity to HU and AZT compared to their parental cells

**Fig. 4:**
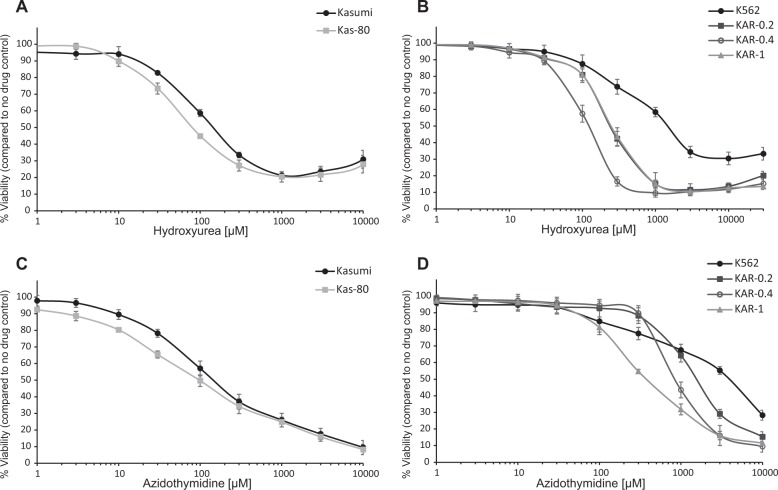
Hydroxyurea (HU) and azidothymidine (AZT) growth inhibition assays. Cells were incubated with increasing HU (**a**, **b**) or AZT (**c**, **d**) concentrations for 72 h and then evaluated for viability using an *XTT cell proliferation kit*. **a**, **c** Dose-dependent growth inhibition of K562, KAR-0.2, KAR-0.4, and KAR-1 cells. **b**, **d** Dose-dependent growth inhibition of Kasumi and Kas-80 cells. Graphs represent the mean results of at least three independent experiments performed in triplicates

Since dCK activity is vital for the NSP, dCK-deficient cells are highly dependent on the DNSP as a source of nucleotides, and could become hypersensitive to DNSP inhibitors such as HU^30^. Based on this knowledge, we tested the growth inhibitory effect of HU on Cytarabine-resistant cells upon a 72 h treatment. We found that all Cytarabine-resistant sublines displayed enhanced sensitivity to HU, as evident from the 2–13-fold decrease in their IC_50_ values, relative to parental cells (all *P*-values ≤ 0.007, Fig. 4a, b and Table 1). Interestingly, this increased HU sensitivity was independent of dCK expression as seen by the similar activity of HU in all K562 sublines. Despite the consistent effects of HU in all Cytarabine-resistant sublines, previous reports indicated that HU is not sufficient for the treatment of resistant/refractory AML in patients^39^. For this reason, we aimed to identify another chemotherapeutic agent to target Cytarabine-resistant cells and possibly achieve a synergistic effect with HU. To this end, we further characterized the NSP in our resistant sublines and detected downregulation of thymidine kinase (TK) 1 in all K562 sublines using WB analysis (Supplementary Fig. S3). This shortcoming in thymidine salvage in the resistant sublines led us to examine their sensitivity to the dCK-independent thymidine antagonist AZT. Growth inhibition assays with AZT showed similar results to HU. While K562 cells displayed intrinsic resistance to AZT with an IC_50_ value > 4 mM (Fig. 4d and Table 1), K562 sublines became gradually more sensitive to AZT with up to tenfold decrease in their IC_50_ values (all *P*-values ≤ 0.0002, Fig. 4d and Table 1). Kasumi cells were considerably more sensitive to AZT than K562 cells (28-fold more sensitive), hence Kas-80 cells had little possibility for change (*P*-value = 0.03, Fig. 4c and Table 1). Notably, both Kasumi and Kas-80 cells displayed very low levels of TK1 protein, similarly to the K562 sublines (Supplementary Fig. S3).

### KAR-1 cells display impaired ɣ-H2AX monoubiquitylation following AZT-induced DNA damage

**Fig. 5:**
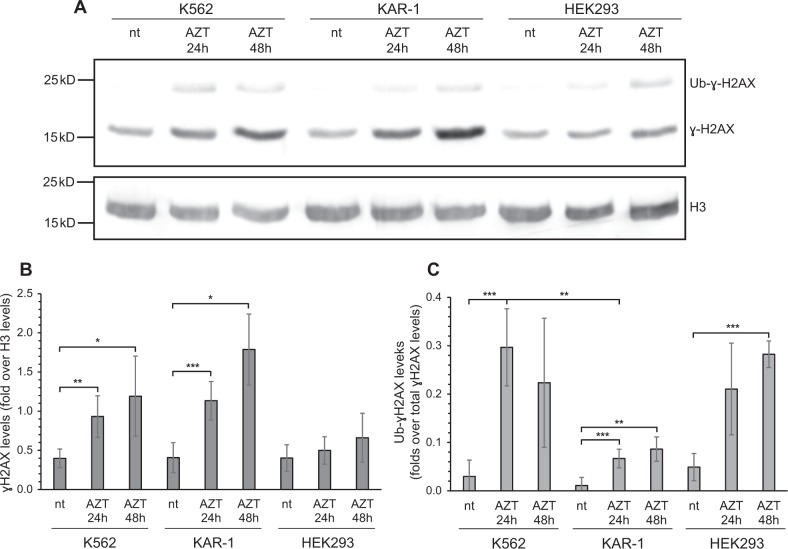
Levels of ɣ-H2AX and monoubiquitylated ɣ-H2AX following AZT treatment. K562, KAR-1, and HEK293 cells were incubated with 500 μM AZT for the indicated times and harvested for chromatin-bound protein extraction. **a** Representative WB analysis of ɣ-H2AX (15 kDa) and monoubiquitylated ɣ-H2AX (ub-ɣ-H2AX, 25 kDa) levels; “nt” represents the drug-free control. **b** Quantification of ɣ-H2AX levels normalized to H3 levels, in three (KAR-1, HEK293) or four (K562) independent experiments. **c** Quantification of ub-ɣ-H2AX levels normalized to total ɣ-H2AX levels, in three (KAR-1, HEK293) or four (K562) independent experiments. Statistical significance is denoted by one (*P*-value ≤ 0.04), two (*P*-value ≤ 0.01), or three (*P*-value ≤ 0.005) asterisks

To further explore the collateral sensitivity to AZT in KAR-1 cells, we followed the levels of histone H2AX Ser139 phosphorylation (i.e., ɣ-H2AX) following 24 and 48 h treatment with 500 μM AZT, compared to parental cells, using WB analysis (Fig. 5). While ɣ-H2AX levels in KAR-1 cells appeared elevated relative to parental K562 cells following 48 h of exposure to AZT (Fig. 5a), potentially indicating increased accumulation of DNA damage, this difference was not statistically significant (Fig. 5b). However, we observed markedly reduced levels of monoubiquitylated ɣ-H2AX in KAR-1 cells compared to K562 following 24 h incubation with AZT (ub-ɣ-H2AX, Fig. 5a, c), suggesting that KAR-1 cells possibly harbor an impaired recruitment of DNA damage repair proteins to AZT-induced DNA damage loci^40,41^.

### The HU and AZT combination results in a synergistic activity in hematopoietic cells but not in epithelial cells

**Fig. 6:**
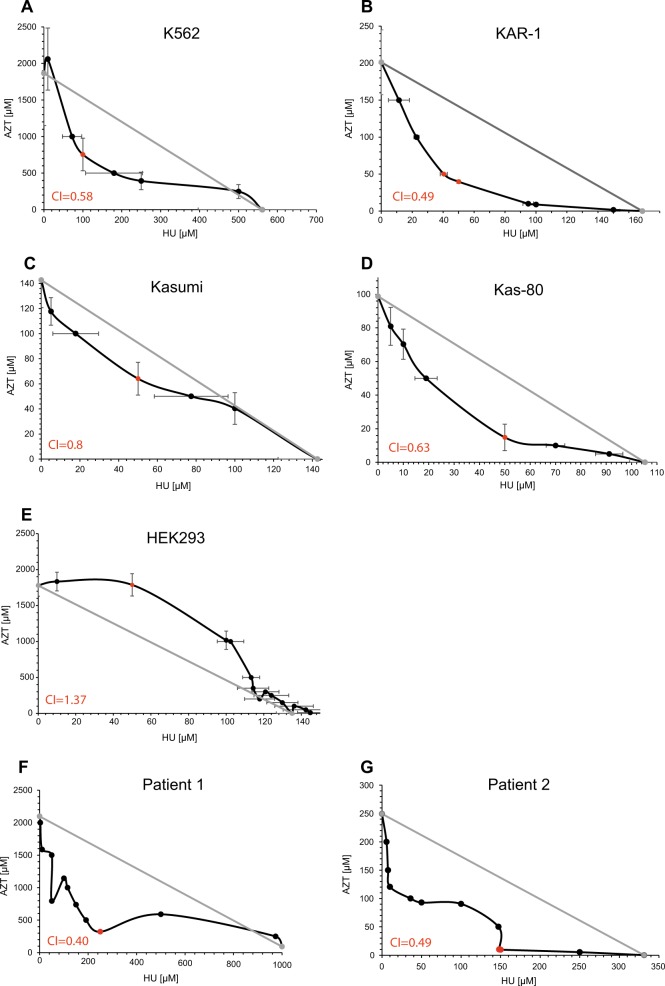
Isobologram analysis of hydroxyurea (HU) and azidothymidine (AZT) combinations. The indicated cell lines (**a**-**e**) or patients' specimens (**f**, **g**) were incubated with a matrix of HU and AZT combinations for 72 h and then evaluated for cell growth inhibition using an *XTT cell proliferation kit*. The graphs display all combinations of drug concentrations resulting in 50% growth inhibition (i.e., IC_50_ pairs); the *Loewe Additivity line*, which connects the axis intersection points (i.e., the IC_50_ value of each drug alone), is displayed in gray. Each experiment was performed at least three independent times in triplicates, excluding the experiments on patients’ specimens (**f**, **g**), which were performed once in triplicates

Recognizing that Cytarabine-resistance uniformly correlated with enhanced HU and AZT sensitivity, and since HU has been previously shown to enhance the cytotoxic effect of AZT^42,43^, we explored the combined cytotoxic capacity of HU and AZT on our sublines. We treated the cells with increasing concentrations of HU and AZT for 72 h as described in the Methods section, and determined the percentage of cell survival compared to untreated cells. The isobologram graph for each cell line showcases the individual drug doses in each IC_50_ pair (drug combinations leading to 50% growth inhibition, Fig. 6). A deep concave isobologram indicated strong synergy between AZT and HU in K562 and KAR-1 cells (Fig. 6a, b); furthermore, comparison of the lowest CI in K562 (i.e., CI = 0.58 for 100 µM HU + 755 µM AZT) and KAR-1 (i.e., CI = 0.49 for 40 µM HU + 50 µM AZT) suggests an enhanced synergistic effect in KAR-1 cells. The AZT-HU synergistic effect in Kasumi cells was less prominent, as evidenced by the IC_50_ pairs that were close to the additivity line and by the lowest CI of 0.8 (for 50 µM HU + 64 µM AZT, Fig. 6c). However, in contrast to the near additive effect in Kasumi cells, HU and AZT achieved a synergistic activity in Kas-80 cells (Fig. 6d) with a lowest CI of 0.63 for 50 µM HU + 15 µM AZT. This further corroborates that the HU + AZT  synergism is intensified following dCK loss and acquisition of Cytarabine resistance. Considering the potential of HU + AZT combination therapy for Cytarabine-resistant AML, we aimed to evaluate the cytotoxicity of this treatment in a model for non-malignant cells, and used HEK293 cells (Fig. 6e). Remarkably, the HU + AZT  combination displayed an antagonistic growth inhibition effect on HEK293 cells, as evidenced from their clear convex graph. This implies that the AZT + HU combination could potentially have a therapeutic window in which it eliminates leukemic cells with residual toxicity to epithelial tissues; for example, a combination of 50 µM HU and 150 µM AZT resulted in growth inhibition of 78 ± 6% in KAR-1 cells, 79 ± 9% in Kas-80 cells, and as low as 15 ± 3% in HEK293 cells. Following these substantial findings in the cell line models, we further expanded this isobologram analysis to white blood cells derived from two AML patients (Fig. 6f–g). While these two specimens displayed distinct responses to the separate drugs (patient 1 exhibited drug resistance, whereas patient 2 was drug sensitive), the combination treatment of HU and AZT was extremely synergistic in both AML specimens, with lowest CI of 0.40 in patient 1 and 0.49 in patient 2. HU alone did not achieve 50% growth inhibition in cells from patient 1, hence the maximal HU concentration (i.e., 1 mM) was used for their additivity line; therefore, their calculated CI is in fact an under-estimation of the extent of synergism. In addition to the isobologram analysis, these primary AML cells were also evaluated for dCK protein levels (Fig. 2i), as well as Cytarabine and DOX^44^ growth inhibition (Table 1). While both patients displayed high dCK protein levels (Fig. 2i), patient 2 exhibited normal sensitivity to Cytarabine and DOX (Table 1), and patient 1 (refractory disease) displayed high DOX and Cytarabine IC_50_ values (Table 1). In agreement with the cell line findings, Cytarabine resistance in patient 1 cells correlated with prominent HU and AZT synergism. However, their normal dCK protein levels (Fig. 2i, lane 1) and their resistance towards four distinctly acting drugs suggested these cells possessed a general anti-apoptotic mechanism rather than an NSP deficiency, thus explaining their lack of collateral sensitivity towards HU and AZT.

## Discussion

**Fig. 7:**
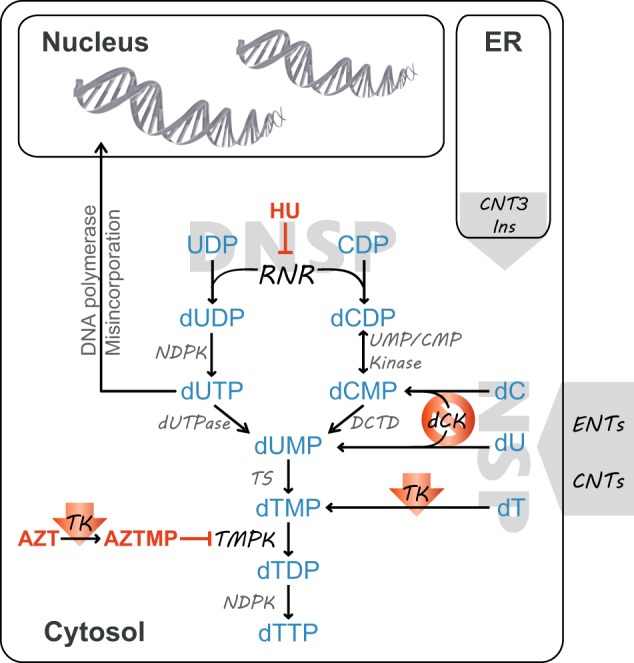
Thymidine metabolism pathways. An illustration of cellular pathways to dTTP synthesis, displaying the steps inhibited by hydroxyurea (HU) or azidothymidine (AZT). NDPK—nucleotide diphosphate kinase; dUTPase—deoxyuridine 5′-triphosphate nucleotidohydrolase; DCTD—deoxycytidylate deaminase; TS—thymidylate synthase; TMPK—thymidylate kinase; dCK—deoxycytidine kinase; TK—thymidine kinase; ENTs—equilibrative nucleoside transporters; CNTs—concentrative nucleoside transporters. Red arrows represent downregulation of TK1, while a red circle represents dCK silencing, in the Cytarabine-resistant sublines

Chemoresistance continues to be a primary hindrance towards curative chemotherapy of multiple cancers^45–49^. Overcoming drug resistance is hence a prime goal of cancer research with paramount importance particularly in malignancies like AML, which are highly drug resistant. The development of antitumor drug resistance may be accompanied by the acquisition of inevitable molecular alterations that may constitute Achilles hill targets that render these chemoresistant tumors highly vulnerable to rationally designed targeted therapies. In this respect, clinical studies have originally confirmed the validity of the novel synthetic lethality concept via targeted inhibition of poly(ADP-ribose) polymerase (PARP) in BRCA1/2-deficient ovarian and metastatic breast cancers^50^. Synthetic lethality describes the relationship between two genes whereby an individual inactivation of either gene results in a viable phenotype, whereas their combined inactivation is lethal. Along this vein, dCK-deficient AML cells lacking NSP may be treated with a proper cytotoxic drug or a drug combination, which targets a central biosynthetic enzyme in the DNSP. Towards this end, we herein identified a markedly synergistic combination of HU and AZT, two clinically approved cytotoxic agents, which efficaciously eradicated human AML sublines displaying >1000-fold resistance to Cytarabine, the anchor drug in AML treatment. Most importantly, this synergistic activity of the HU and AZT combination against AML cells was potentiated upon acquisition of Cytarabine resistance. In contrast, this drug combination exhibited a *bona fide* antagonistic activity towards normal HEK293 cells, suggesting that normal tissues may be intrinsically shielded from this otherwise cytotoxic drug combination. Furthermore, this synergistic activity was demonstrated on primary AML patient specimens, thus providing an initial proof of concept warranting a wide scale ex-vivo study towards future clinical trials. Consistent with our present findings, HU and AZT, which are both independent of dCK activity status were previously found to elicit a synergistic cytotoxic activity against human acute lymphoblastic and chronic myelogenous leukemia cell line models^42,43^. Hence, the inevitable question that emerges from our present findings concerns the molecular mechanism underlying the potent synergistic activity of this drug combination against Cytarabine-resistant AML cells. As depicted in Fig. 7, we propose that the synergistic activity of HU and AZT is based upon their shared deleterious impact on depletion of cellular dTTP pool. Given that dTMP is a common product of both NSP and DNSP, inhibition of TMPK by azidothymidine monophosphate (AZTMP)^51,52^ will necessarily disrupt both salvage and de novo pathways of dTTP generation; this will culminate in depletion of dTTP pools and consequent misincorporation of dUTP into DNA^53,54^. Although this blockade of TMPK by AZT cannot be bypassed, it could be alleviated by competition with the accumulated dTMP. As to HU, in addition to enhancing the cytotoxic effect of AZT by increasing its phosphorylation^55^, its potent inhibition of RNR also disrupts dTMP generation, thereby potentiating dTTP pool depletion inflicted by AZT. In this respect, cells with intact NSP, including K562 can be rescued via enhanced thymidine uptake and salvage pathway. In contrast, our findings suggest that exposure of Cytarabine-resistant cells, which are uniformly devoid of NSP due to dCK and TK1 downregulation, to the HU + AZT combination, would result in a complete blockade of dTTP synthesis. Hence, these cells would be deprived of both salvage and de novo nucleotide biosynthesis pathways, resulting in a synergistic activity. In support of this proposed mode of action of the HU + AZT combination is our finding that upon exposure of Cytarabine-resistant cells to AZT, they displayed decreased nuclear levels of monoubiquitylation of Ɣ-H2AX, which is necessary for elicitation of the DNA damage response, hence highlighting their susceptibility to DNA damage inflicted by AZT^40,41^.

Expansion of hematopoietic clones, which acquired a genetic trait(s) that confers upon them a survival advantage under chemotherapeutic drug treatment, is a well-established phenomenon underlying AML recurrence^56,57^. Consistently, a major determinant of Cytarabine resistance emerging from the current study is the targeted molecular mechanisms of loss of dCK function. Specifically, during the establishment of Cytarabine-resistant AML sublines, we identified a novel mechanism of stable dCK inactivation via introduction of a 5 bp genomic deletion resulting in a frameshift, hence provoking dCK transcript degradation via NMD. Since this deletion was present in all advanced Cytarabine-resistant K562 sublines, it is remarkable that a clone harboring this 5 bp frameshift deletion emerged at an early stage of Cytarabine selection (as seen in KAR-0.2 cells), underwent clonal expansion and became a dominant and genetically stable trait, which conferred high level resistance to Cytarabine. It should be noted that upon an extended culturing of low-level resistant KAR-0.2 cells in drug selective medium with no further increase in Cytarabine concentrations, dCK silencing and loss of dCK protein occurred. Thus, stable genetic inactivation of the dCK gene, loss of transcription and protein expression are a dominant genetic trait, which confers >1000-fold resistance to Cytarabine. We also identified a previously described mechanism of loss of dCK function based on genomic deletion of the entire dCK locus. In this respect, whereas Kas-80 cells displayed a homozygous deletion specific to the dCK locus, Cytarabine-resistant K562 sublines exhibited variable genomic dCK losses, indicating a heterozygous dCK deletion and/or a heterogeneous drug-resistant cell population. These findings are in accord with previous literature regarding the role of dCK loss of function in Cytarabine resistance in AML.

Whereas advanced stages of acquisition of Cytarabine-resistance were uniformly based on loss of dCK function in both K562 and Kasumi sublines, the early stage of Cytarabine-resistance represented by the KAR-0.2 subline, fully retained parental dCK protein levels. Thus, the question that emerges is what are the putative dCK-independent mechanisms of resistance to Cytarabine in the KAR-0.2 subline? While a definitive mechanism was not identified, we note that the transcripts of CNT3 and CDA were overexpressed 80-fold relative to parental cells; however, the CDA protein was neither expressed in parental cells nor in their KAR-0.2 subline. Moreover, CNT3 should be downregulated in order to contribute to Cytarabine resistance, and prolonged growth in stable drug selective conditions (0.2 µM Cytarabine) resulted in a marked fall in CNT3 mRNA levels, yet still higher than parental levels. Although the primary localization of CNT3 is the plasma membrane where it acts as a concentrative nucleoside transporter, recent studies have shown that its novel splice variant CNT3-Ins localizes to the ER^33^. It has been postulated that CNT3-Ins may function in the salvage of nucleotides from the ER lumen to the cytoplasm; this could possibly elevate the cytoplasmic nucleoside pool to negate the cytotoxic activity of Ara-CTP nucleotides. In contradistinction, a previous study has shown that higher gene expression levels of CNT3 were found in AML patients harboring the t(8:21) cytogenetic abnormality. Increased CNT3 gene expression was associated with favorable outcomes and longer disease-free survival in AML patients^58^. Clearly, in the absence of an available antibody to CNT3, this putative ER retention modality and its possible role in conferring Cytarabine-resistance cannot be addressed experimentally at the present time. Notably, our current study demonstrates that the mere use of biomarkers for disease prognosis and patient outcome in the form of transcript levels such as CNT3 and CDA without corroboration of their actual protein levels could be highly misleading as exemplified by the 80-fold increase in CDA gene expression in KAR-0.2 cells, while the protein was completely undetectable.

## Supplementary information

Supplementary Information
