# Supplementary material for: Surmounting Cytarabine-resistance in acute myeloblastic leukemia cells and specimens with a synergistic combination of hydroxyurea and azidothymidine
Source: Cell Death Dis. 2019 May 17;10(6):390. doi: 10.1038/s41419-019-1626-x (PMC6525253; doi:10.1038/s41419-019-1626-x)

Supplementary Information

"Surmounting Cytarabine-resistance in acute myeloblastic leukemia cells and specimens with a synergistic combination of hydroxyurea and azidothymidine" by May Levin, Michal Stark, Bluma Berman and Yehuda G. Assaraf, Cell Death & Disease (2019).

Contents:

Supplementary Table 1

Supplementary figure legends

Supplementary Figure S1

Supplementary Figure S2

Supplementary Figure S3

Supplementary Table 1: Primers for PCR and sequencing (1-10), and RT-PCR (11-34)

	Primer	Sequence
1	dCK F-EX1	CCAAAGTCAAACCCCGACAC
2	dCK R-Int1	ATGCTAAATGAAAGACGCCAGA
3	dCK F-EX3	GAACTTACAATGTCTCAGAAAAATGG
4	dCK R-EX4	CATTCAGATTCATACAAATTAGATGC
5	dCK F-EX4	GCATCTAATTTGTATGAATCTGAATG
6	dCK F-EX5	ACATGCTTACATAGAATATATTTACGG
7	dCK R-EX6	CTTTTCAACCAGACTTTCATATTTG
8	dCK R-EX7	GAACCATTTGGCTGCCTGTAG
9	dCK F-3UTR	CTACAGGCAGCCAAATGGTTC
10	dCK R-3UTR	TGGATGCTTTCTAGCCTCTGTC
11	dCK RT 1F	GCCGCCACAAGACTAAGGAA
12	dCK RT 2R	GACTTCCCTGCAGCGATGTT
13	gdCK RT F	CTCACTAGCTGACCCGGCA
14	gdCK RT R	GCTCCTTACCGATGTTCCCT
15	ENT1 RT F	GGGCAGCCTGTTTGGTCT
16	ENT1 RT R	CCACTGGCAATAGCGCAG
17	ENT2 RT F	CTCCTGTCCATGGCCAGTG
18	ENT2 RT R	GGGCCTGGGATGATTTATTG
19	ENT3 RT F	TCAGCGGTGCCTCCACTGT
20	ENT3 RT R	GCAGCCAAGTCCACCAATGA
21	CLRN2 RT F	TCACGACCTGACGGAACGA
22	CLRN2 RT R	GTTGTGAACAGGGGAAGGCT
23	Shroom3 RT F	CGGCTTACAGCTCTTTCTCCA
24	Shroom3 RT R	CCCTCCGGGTGTCATAGACTG
25	CNT3 RT F	ACATTTCTTTTGGGGTTCCAT
26	CNT3 RT R	GCAATCAGATTCACAGCGATG
27	CNT3 Ins RT F	GCTCCACCTCCATCTATTTCCT
28	CNT3 Ins RT R	TTTTGTTGCATCTCCTCATCATC
29	CDA RT F	TGTGCTGAACGGACCGCTA
30	CDA RT R	GCAGGTCCTCAGGCCCAA
31	GUSB RT F	CCATTCCTATGCCATCGTG
32	GUSB RT R	ATGTCGGCCTCGAAGGG
33	gFR-α RT F	CTGGAGCCCTGCACACAACTTA
34	gFR-α RT R	GCTCATGCAACTTGTCCTCGG

Supplementary Figure legends

Figure S1: Cytarabine selection process of K562 cells and an exemplifying timeline of KAR-0.2 cells continuously growing in selection. K562 cells were grown in 200 nM Cytarabine for one month until regaining their doubling time. The established subline (termed KAR-0.2) was either continuously grown in 200 nM Cytarabine, grown in drug-free medium to establish KAR-0.2(-) cells, or transferred to grow in 400 nM/1 μM Cytarabine for a month to establish KAR-0.4 and KAR-1 cells, respectively. KAR-1 cells were either continuously grown in 1 μM Cytarabine or transferred to grow in drug-free medium to establish KAR-1(-) cells. The table exemplifies Cytarabine IC_{50} values and dCK mRNA expression of KAR-0.2 cells throughout a continuous growth period in 200 nM Ara-C.

Figure S2: Expression of genes related to Cytarabine metabolism. Cells at mid-log phase were harvested for RNA purifications (A-D), or cytosolic (cp) and nuclear (np) protein extractions (E). RT-PCR analysis was used to evaluate mRNA expression of ENT1-3 (A), CNT3 (C), and CDA (D) in K562, KAR-0.2, KAR-0.4 and KAR-1 cells, as well as the expression of ENT1-3 and CNT3 in Kasumi and Kas-80 cells (B). CNT3-Ins represents a CNT3 splice isoform with ER localization. WB analysis was used to evaluate CDA protein expression in K562 and KAR-0.2 cells (E) compared to HeLa cells, using CRT as loading control.

Figure S3: TK1 protein expression. Cells at the mid-log phase were harvested for cytosolic protein extractions, and analyzed for TK1 expression using WB analysis with CRT as loading control. Relative intensities of the TK1 band were calculated in comparison to K562 using the *Image J* software. Showcased are two representative depictions of TK1 protein levels in K562 sublines (A, B) and in Kasumi and Kas-80 cells (B). Noticeably, KAR-1 cells displayed variable TK1 levels ranging from 7-36% of K562 throughout seven repeats (represented in panels A and B, respectively).

Supplementary Figure S1

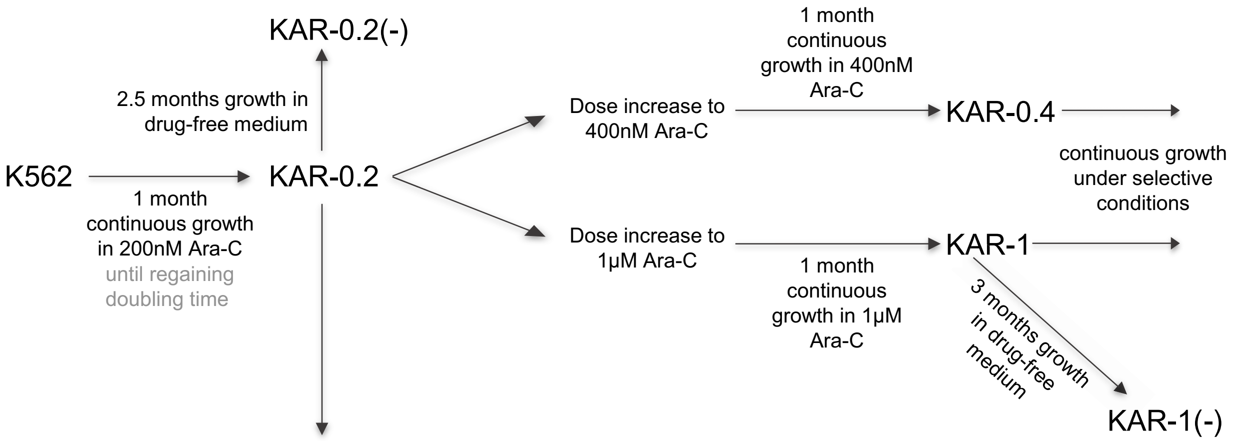

KAR-0.2 Timeline during continuous growth under selection:

Days from Establishment	12	14	19	20	24	30	34	38	44	77	78	84	129
Ara-C IC50 [μM]	-	2.8	-	-	8	14.9	82.7	>100	-	>100	-	>100	-
dCK mRNA expression in RT-PCR (%of K562):	136	-	143	104	-	58	-	-	30	18	20	26	6

Supplementary Figure S2

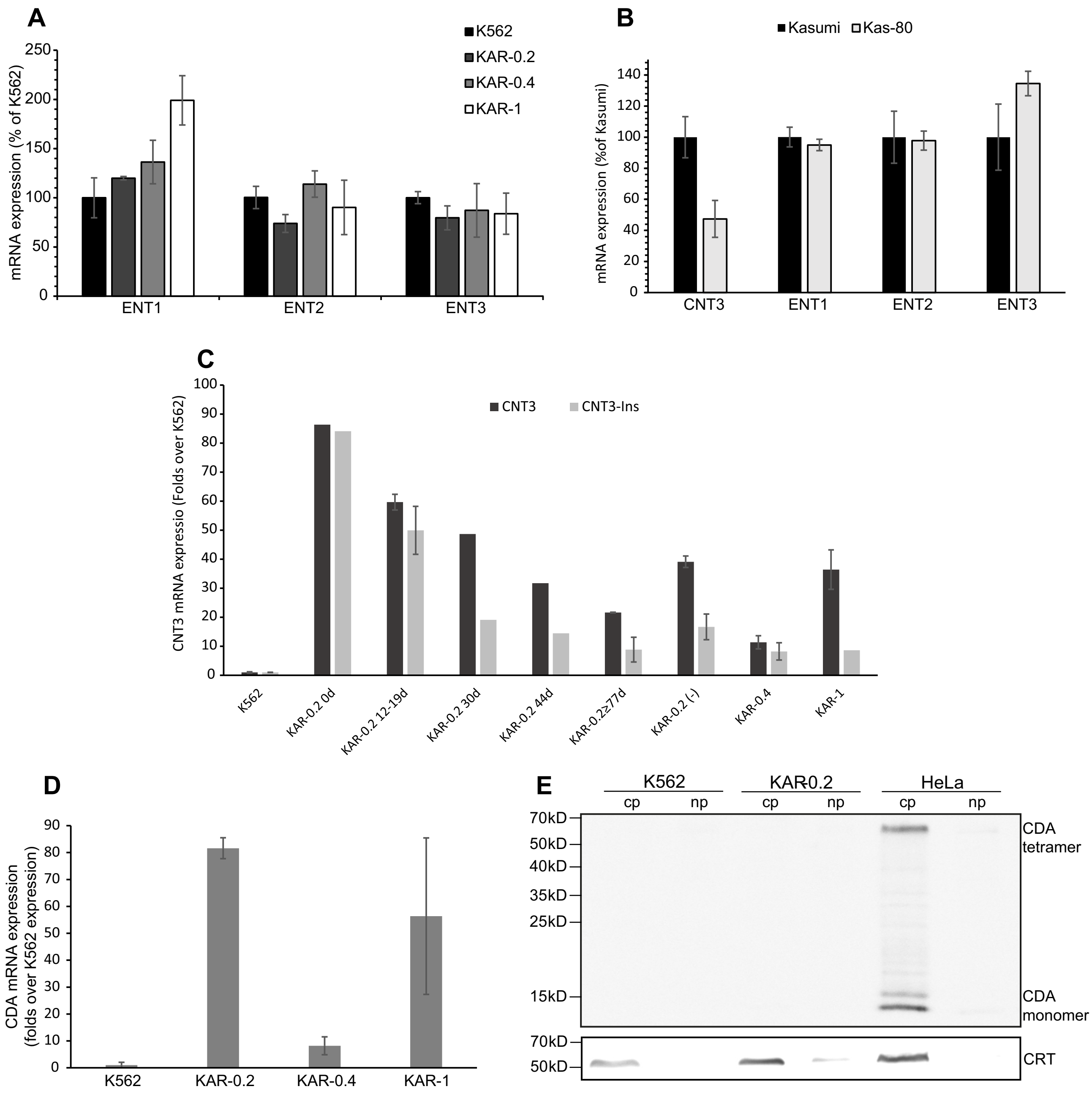

Supplementary Figure S3

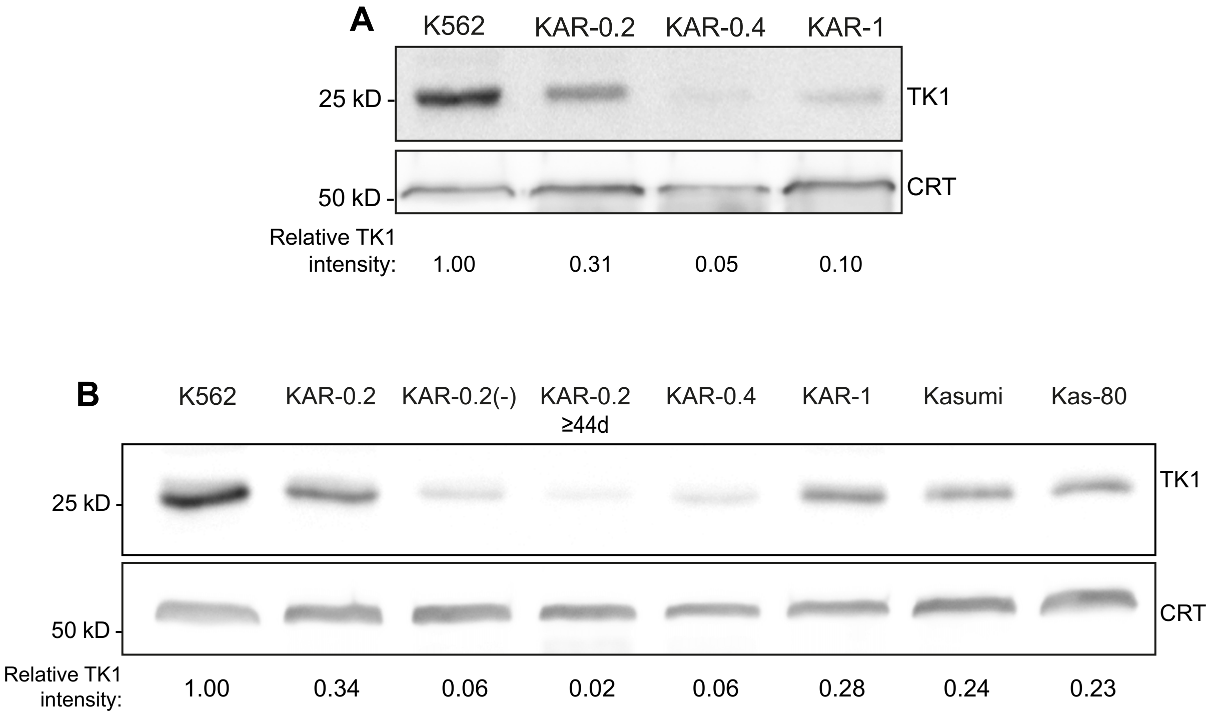

Supplement: Supplementary file 1 — Supplementary Information [file 41419_2019_1626_MOESM1_ESM.pdf]
